# Hyperemesis Gravidarum in First-Trimester Pregnant Saudi Women: Is *Helicobacter pylori* a Risk Factor?

**DOI:** 10.3389/fphys.2020.00575

**Published:** 2020-06-24

**Authors:** Khulood S. Hussein

**Affiliations:** Faculty of Medicine, Department of Physiology, King Abdulaziz University, Jeddah, Saudi Arabia

**Keywords:** hyperemesis gravidarum, *Helicobacter pylori*, pathogenesis, early pregnancy, Saudi women

## Abstract

**Introduction:**

Hyperemesis gravidarum (HG) is a serious complication of pregnancy involving nausea and vomiting which affects all facets of the lives of many women. *Helicobacter pylori* infection has been linked to HG in some regions of the world. However, the prevalence of *H. pylori* in Saudi Arabian pregnant women and its link to HG has not been the subject of previous research. Detecting and treating *H. pylori* infection in women early in their pregnancies may lower the likelihood of adverse maternal outcomes. This study aims to assess the connection between the pathogenesis of HG and *H. pylori* infection in this population.

**Methods:**

Forty-five pregnant women with HG were recruited from the outpatient clinic for antenatal care in the Gynecology and Obstetrics Department at King Abdulaziz University Hospital. Forty-five pregnant women without HG were matched as controls. Both groups underwent testing for the *H. pylori* antigen in stool samples.

**Results:**

A statistically significant difference (*P* < 0.05) was observed between the cases and controls in terms of the occurrence of *H. pylori*. Thirty-eight women in the HG group (84.4%) tested positive for *H. pylori*, while the same was true of only 20 of the controls (44.4%). The mean level of blood hemoglobin in positive cases was significantly lower than that in negative cases (9.56 ± 1.29 vs. 11.90 ± 1.18 g/dl, *P* = 0.012).

**Conclusion:**

*H. pylori* may play a contributing role in the presence of HG in the study population. It may be included with other investigations of HG, especially with cases that do not respond to conventional management and continue into the second trimester. Women with *H. pylori* were also more likely to suffer from anemia compared to those without the infection. For this reason, those working with pregnant women should pay close attention to those infected with *H. pylori*. Additional large case–control studies are necessary to better understand the part *H. pylori* plays and the pathogenesis of HG.

## Introduction

Hyperemesis gravidarum (HG), a disorder characterized by ongoing severe nausea and vomiting with resulting ketosis, affects 0.3–2% of women during pregnancy (McCarthy et al., 2014). Those affected lose over 5% of their prepregnancy weight. Hyperemesis gravidarum can also result in dehydration, nutritional deficits, ketonuria, ketonemia, irregularities in the electrolyte, and acid–base balance, and, in the most severe cases, even death (Goodwin, 2008). Dehydration and disruption of the acid–base balance may result in liver and kidney damage (Eliakim et al., 2000). The condition usually starts in early pregnancy between 4 and 9 weeks of gestation and reaches a peak at 12–15 weeks, with resolution usually occurring at 20 weeks of gestation (Niebyl, 2010). However, a considerable number (9–20%) of women presenting with HG report continuity of nausea and vomiting beyond 20 weeks of gestation that can persist till the end of pregnancy (Ebrahimi et al., 2009). Occurring in 0.3–1.5% of all live births, HG is one of the foremost reasons for hospitalization during the earliest 3 months of pregnancy (Verberg et al., 2005). Hyperemesis gravidarum can debilitate the lives of women who suffer from it. If the condition is not properly managed, it can result in considerable health problems, including imbalances in electrolytes, malnourishment, Wernicke’s encephalopathy, clotting, depressive disorders, and adverse gestational outcomes such as prematurity, undersized fetuses, impaired development, and fetal anomalies (Dodds et al., 2006).

Many factors appear to play a role in the etiology of HG (Eliakim et al., 2000). Contributing to the pathologic mechanism triggering HG are the following: gastrointestinal tract disorders, nutritional deficiencies, endocrine factors, genetic incompatibility, and immunological factors. Some research has implied that *Helicobacter pylori* infection during pregnancy may be implicated in HG, but the data are not conclusive (Golberg et al., 2007). One suggestion is that the decrease in the production of stomach acid in early pregnancy stemming from a rise in accumulated body fluid, immunologic tolerance, and steroid hormone changes could activate dormant *H. pylori* infection, thereby worsening nausea and vomiting (Kocak et al., 1999). Recent work has shown that placentation, appetite, and cachexia genes *GDF15* and *IGFBP7* are linked to HG (Fejzo et al., 2018a, b, 2019). However, no theory on its own adequately explains HG (Dodds et al., 2006; Eliakim et al., 2000; Verberg et al., 2005).

*Helicobacter pylori* infection can be found in roughly 50% of the global population, with higher rates in developing countries (Malaty, 2007). *H. pylori* are a type of bacteria colonizing the stomach, usually in childhood. The resulting asymptomatic infection can be chronic (Suerbaum and Michetti, 2002). In a small number of people with *H. pylori*, peptic ulcers and gastric carcinoma develop, typically later in adulthood (Suerbaum and Michetti, 2002). With pregnancy comes a rise in the risk of *H. pylori* (Lanciers et al., 1999), and the rate of *H. pylori* infection in pregnant women differs based on geographical region and socioeconomic conditions. Even the technique used for *H. pylori* testing can affect *H. pylori* prevalence. Various mechanisms have been suggested to explain the link between *H. pylori* infection and undesirable effects during pregnancy. These include anemia, low blood platelet count, intrauterine fetal growth restriction, and miscarriage (Cardaropoli et al., 2014). A number of recent studies have highlighted the relationship between infection with *H. pylori* and the risk of HG (Frigo et al., 1998; Bezircioglu et al., 2011; Guven et al., 2011; Mansour and Nashaat, 2011). Previous studies have recommended further examination of *H. pylori* infection as a potential contributor to iron deficiency anemia seen in pregnant women (Muhsen, 2013).

Treatment of *H. pylori* infection is becoming increasingly important, with a corresponding urgency for screening methods that are straightforward, accurate, cost-effective, and non-invasive. Ethical concerns restrict the use of invasive diagnostic techniques on pregnant women, and urea breath tests are out of the question because of the use of radioactive materials (Azami et al., 2017). Instead, it is recommended that serologic and stool antigen tests be used as a fast non-invasive way to initially screen for *H. pylori* infection. Serologic screening works by detecting the presence of anti-*H. pylori* IgG antibodies in serum. However, both current and previous *H. pylori* infections appear the same in this type of screening. The accuracy of diagnosis also varies among the commercial kits available (range, 68–82%) (Feldman et al., 1995). Stool antigen screening involves an enzyme immunoassay that can diagnose the active infection with *H. pylori* by detecting the antigen in stool samples. With its accuracy and non-invasive nature, the stool test is thus recommended as a way to screen for *H. pylori* (Gisbert et al., 2006; Azami et al., 2017).

While studies have been done showing the hyperendemic nature of *H. pylori* infection among various groups in Saudi Arabia, none of the research focuses on *H. pylori* infection in expectant women (Akeel et al., 2018). The multifaceted nature of maternal health means various studies are needed to clarify the factors involved in *H. pylori* infection among pregnant women. Several recent studies of *H. pylori* in various populations have seen a significantly high rate of the infection among pregnant women who have HG (Frigo et al., 1998; Gisbert et al., 2006; Mansour and Nashaat, 2011). Research on *H. pylori* infection in expectant Saudi women is scarce, so this study aims to assess the incidence and the possible association between *H. pylori* infection and HG.

## Subjects and Methods

In Saudi Arabia, health care coverage is universal and free for all citizens and legal residents. The Ministry of Health and other governmental health departments provide 80% of health services, while the private health sector provides the remainder (Almalki et al., 2011). Maternal health care services in Saudi Arabia include extensive prenatal care. Expectant women receive important health education, and their histories are taken. In addition, they are screened for conditions like anemia and hypertensive disorders. Screening, preventing, and managing infectious diseases are also part of prenatal care, as is the provision of prophylactic medication (The Partnership for Maternal Newborn and Child Health, 2011). Such prenatal care is widely available in Saudi Arabia, beginning in the first trimester, providing at least eight visits for women who have pregnancies without complications (Alanazy and Brown, 2020). This case–control study was conducted on 45 expectant women in their initial 3 months of pregnancy presenting with HG at King Abdulaziz University (KAU) Hospital of Gynecology and Obstetrics (a tertiary hospital), Jeddah, Saudi Arabia, from January 2017 to May 2017 and compared them with the same number of healthy pregnant women as a control group. Women in the case group were matched with women in the control group for maternal and gestational ages. The KAU biomedical ethical committee gave its approval for the study, and all women gave informed consent.

Women were classified as having HG if they displayed all of the following conditions: severe nausea and vomiting happening at least thrice daily; weight reduction of 5% of body weight since symptoms started; and evidence of at least one positive ketonuria. A comprehensive history was obtained from each woman by the general physician, which included her history of medical conditions and the use of medications for ongoing conditions. The histories also served to exclude participants with overactive thyroid, psychological disorders, liver conditions, urinary tract infection, and intracranial abnormalities. Following general and localized examinations, any associated medical disorders such as thyroid diseases, pyelonephritis, pancreatitis, peptic ulcer, and diabetes mellitus were excluded. Both groups of women then underwent ultrasound scanning to assess gestational age and rule out a misdiagnosis of HG due to other conditions such as twin pregnancy, molar pregnancy, or missed miscarriage.

Blood samples were drawn from all eligible subjects enrolled in the study for biochemical analysis including hemoglobin (Hb), hematocrit (Hct) value, hepatic enzymes [alanine aminotransferase (ALT) and aspartate aminotransferase (AST)], and renal functions (serum urea and creatinine).

### Determination of *H. pylori* Antigens in Stool Specimens

The presence of *H. pylori* antigens in the stool specimens was assessed using the rapid, one-step *H. pylori* card test (H + R *H. pylori* CARD, Madrid, Spain), which is a chromatographic immunoassay used to diagnose *H. pylori* infection (Calik et al., 2016). On the test band region, monoclonal antibodies are used to precoat the membrane against *H. pylori* antigens. Assessment involves allowing a reaction between the sample and the colored conjugate (anti-*H. pylori* monoclonal antibodies–red polystyrene microspheres) which has been pre-applied to the test strip. Capillary action then pushes the mixture up the membrane, causing the colored particles to migrate. The capture of the colored conjugate by the specific antibodies on the membrane indicates a positive result. The mixture continues its passage across the membrane to the antibody that is immobilized in the control band area. At this point, a red band emerges, indicating an internal control for the reagents and confirmation of proper flow and adequate volume.

Participants’ stool samples were tested following instructions from the test card manufacturer. Tests were deemed negative if one red band appeared in the middle area in the region with the control line. Tests were deemed positive if a red band appeared in both the result band and the control band. Tests without a red line in the control area were deemed invalid, irrespective of the result site’s appearance.

### Statistical Analysis

Statistical analysis was carried out with the use of SPSS version 17.0 for Windows (SPSS Inc., IL, United States). Continuous variables were reported as mean ± standard deviation or median (range), as appropriate. Categorical variables were described using frequency distributions and were reported as the frequency [*n* (%)], compared using chi-square and Fisher’s exact test. Student *t* and Mann–Whitney *U* tests were applied for a comparison of mean scores of continuous variables between the HG group and women in the control group. Statistical significance was set at a *P*-value < 0.05.

## Results

Table 1 illustrates the demographic data of the study population. The maternal age of the women in the case group ranged from 18 to 37 (mean 26.46 ± 5.68), and in the control group, the maternal age ranged from 20 to 36 (mean 28.07 ± 4.85). The independent-sample *t*-test showed no significant differences between the mean ages of cases and controls (*t* = -1.443, *P* = 0.153). The difference between controls and cases in terms of calculated gestational age, body mass index (BMI), and parity distribution was not significant.

**TABLE 1:** Sociodemographic characteristics among the two groups.

Parameter	Control (*n* = 45)	Case (*n* = 45)	*P*-value
	Mean ± SD	Mean ± SD	
Maternal age (years)	28.07 ± 4.85	26.46 ± 5.68	NS
Gestational age (weeks)	10.60 ± 2.15	10.50 ± 2.46	NS
BMI	22.33 ± 1.21	22.62 ± 1.11	NS
	**Number (%)**	**Number (%)**	
Nulliparity	23 (51.1)	27 (60)	
Primiparity	13 (28.9)	10 (22.2)	NS
Multiparity	9 (20)	8 (17.8)	

*Non significant.*

As shown in Table 2, the mean levels of blood Hb were significantly lower in cases than in controls (10.56 ± 1.20 vs. 11.30 ± 1.33, *P* = 0.004). Similarly, sodium and potassium serum electrolyte levels were significantly reduced in cases compared to those in controls (*P* < 0.05). However, the Hct value displayed a significant increase in women with HG as opposed to women without the condition (*P* < 0.05).

**TABLE 2:** Hematological and biochemical characteristics among cases and controls.

Parameter	Control (*n* = 45)	Case (*n* = 45)	*P*-value
	Mean ± SD	Mean ± SD	
Hb (g/dl)	11.30 ± 1.33	10.56 ± 1.20	0.004*
Hct (%)	32.50 ± 3.58	34.18 ± 4.01	0.037*
ALT (U/l)	19.30 ± 6.64	33.15 ± 7.70	0.001*
AST (U/l)	18.70 ± 7.47	31.63 ± 6.44	0.001*
Urea (mg/dl)	19.93 ± 5.63	17.30 ± 6.17	0.038*
Creatinine (mg/dl)	0.72 ± 0.14	0.65 ± 0.16	0.031*
Na^+^ (mEq/l)	135.3 ± 4.45	133.09 ± 5.432	0.038*
K^+^ (mEq/l)	5.06 ± 1.07	3.43 ± 0.58	0.05*

* Significant difference at *P* < 0.05.

The activities of serum AST and ALT as marker enzymes of liver function as well as the levels of serum urea and creatinine as markers of kidney function are shown in Table 2. There were significant elevations in ALT and AST activities in cases compared to those in controls (33.15 ± 7.70 and 31.63 ± 6.44 U/l vs. 19.30 ± 6.64 and 18.70 ± 7.47 U/l, *P* = 0.001). In contrast, urea and creatinine concentrations showed significantly decreased activities in cases compared to those in controls (17.30 ± 6.17 and 0.65 ± 0.16 mg/dl vs. 19.93 ± 5.63 and 0.72 ± 0.14 mg/dl, *P* < 0.05).

The distribution of the *H. pylori* stool antigen (HpSA) among the study population is presented in Table 3. Thirty-eight (84.4%) cases tested positive for the HpSA compared to 19 (42.2%) controls. There was a significant difference between the two cohorts (χ^2^ = 8.561, *P* = 0.003), with a higher distribution of HpSA among cases.

**TABLE 3:** Distribution of *H. pylori* stool antigen (HpSA) among the two groups.

HpSA	Control (*n* = 45)	Case (*n* = 45)	*P*-value
	*N* (%)	*N* (%)	
Positive (+)	19 (42.2)	38 (84.4)	0.003*
Negative (−)	26 (57.8)	7 (15.5)	

* Significant difference at *P* < 05.

Table 4 indicates the relationship between *H. pylori* and maternal age, gestational age, hematological parameters, liver and kidney functions, and electrolyte parameters studied in the case group. The results showed no significant difference in positive compared to negative cases (*P* = not significant) in all studied parameters. However, the mean level of blood Hb in positive cases was significantly lower than that in negative cases (9.56 ± 1.29 vs. 11.90 ± 1.18 g/dl, *P* = 0.012). Conversely, compared to the AST activity in cases testing negative to *H. pylori*, that in cases testing positive was significantly elevated (32.45 ± 8.01 vs. 27.54 ± 7.23 U/l, *P* = 0.045).

**TABLE 4:** Relationship of *H. pylori* to different parameters among cases.

Parameter	*Helicobacter pylori*		*P*-value
	Positive (*n* = 38)	Negative (*n* = 7)	
	Mean ± SD	Mean ± SD	
Age (years)	27.06 ± 5.47	27.69 ± 5.03	NS
Gestational age (weeks)	10.06 ± 2.74	10.52 ± 2.09	NS
Hb (g/dl)	9.56 ± 1.29	11.90 ± 1.18	0.012*
Hct (%)	33.92 ± 3.92	33.52 ± 3.45	NS
ALT (U/l)	34.09 ± 9.70	31.50 ± 7.36	NS
AST (U/l)	32.45 ± 8.01	27.54 ± 7.23	0.045*
Urea (mg/dl)	18.19 ± 5.83	19.58 ± 6.49	NS
Creatinine (mg/dl)	0.68 ± 0.14	0.71 ± 0.18	NS
Na^+^ (mEq/l)	134.3 ± 4.87	133.8 ± 5.60	NS
K^+^ (mEq/l)	3.60 ± 0.70	3.55 ± 0.52	NS

* Significant difference at *P* < 0.05. NS; Non significant.

## Discussion

The present findings documented a significantly higher occurrence of *H. pylori* in pregnant women who suffer from HG than in women with normal pregnancies (84.4 vs. 42.2%; *P* = 0.003). As with our findings, numerous studies have established a significant positive correlation between HG and the presence of *H. pylori* (Frigo et al., 1998; Jacoby and Porter, 1999; Kocak et al., 1999; Cevrioglu et al., 2004; Gisbert et al., 2006; Mansour and Nashaat, 2011).

In their study of expectant women with HG, Abd Alwahed et al. (2014) found that these women have a significantly higher *H. pylori* prevalence than those who do not have HG (69 vs. 15%; *P* < 0.001). In a systematic review that involved 1,732 participants and controls, Golberg et al. (2007) observed more cases of HG in pregnant women infected with *H. pylori* compared to those without *H. pylori* (pooled OR = 4.45; 95% CI: 2.31–8.54).

Bezircioglu et al. (2011) reported significantly more cases of *H. pylori* infection in pregnant women presenting with HG than in those without the condition (22.2 vs. 2.8%; *P* = 0.037) (Lanciers et al., 1999). Similarly, recent investigations of different populations showed a significantly high prevalence of expectant mothers with HG testing positive when screened for *H. pylori* (Al-Basam and Obaid, 2014; Guven et al., 2011). Shirin et al. (2004) also found that women with first-trimester vomiting had a higher likelihood of being infected with *H. pylori* (81.2 vs. 65%, *P* = 0.0004).

In their study of pregnant women who have HG, Güngören et al. (2013) divided the subjects into three groups according to the severity of their symptoms: mild, moderate, and severe. They found that 21 (87.5%) of the participants with severe HG symptoms had an elevated *H. pylori* positivity rate (*P* = 0.001), suggesting that HG positively correlates with *H. pylori* infection.

In their meta-analysis of the *H. pylori*–HG relationship, Li et al. (2015) examined studies published in various databases published before March 20, 2014. Their analysis pointed to a significantly higher occurrence of *H. pylori* infection in expectant women who have HG (*P* < 0.001). An examination of subgroups also suggested infection with *H. pylori* as a risk factor for HG in Africa in particular, but this was also the case in Asia and Oceania (*P* < 0.001). This systematic review suggests that *H. pylori* is regarded as one of the risk factors for HG, in particular in developing nations.

On the other hand, other studies failed to show significant association or found only a weak association between HG and *H. pylori*. For instance, Lee et al. (2005) reported a slightly lower rate of *H. pylori* in Hispanic American women with HG in comparison to those not suffering from the condition (65 vs. 67%, *P* = not significant) (Lee et al., 2005). Similarly, in Turkey, Karadeniz et al. (2006) reported reduced rates of *H. pylori* in women with HG than in those without (68 vs. 79%, *P* = not significant). Aytac and Kanbay (2007) found no significant difference in *H. pylori* rates between pregnant women with HG and controls (41.1 vs. 40%, respectively), indicating that HG is not associated with *H. pylori* infection. In a study of immigrant women in Norway, Vikanes et al. (2013) reported no significant correlation between severe HG symptoms and exposure to *H. pylori*. These contradictory findings likely stem from the lack of a universally accepted definition of HG, suggesting a wide variation in the study population.

In their case–control study of 80 subjects (40 in each group), Guven et al. (2011) found that the mean maternal age of women with HG and HpSA positivity and women in the control group to be significantly different (25.8 vs. 28.4 years, respectively, *P* = 0.025). Although this suggests that age is a noteworthy risk factor for *H. pylori* infection in women who have HG, our results suggest something different. Our findings did not confirm any association between HpSA positivity and maternal age and are thus in accordance with previous studies (Bezircioglu et al., 2011; Abd Alwahed et al., 2014). We found no significant variation in maternal age between women with HG and those without, which may be explained by the limited range of reproductive age of our study participants. Besides age, other theories also exist regarding the pathogenesis of HG, including social factors observed in patients with HG (Karac et al., 2004).

Gestational anemia is a public health concern and is particularly problematic in the developing world. Its association with unfavorable outcomes in pregnancy is well known (Black et al., 2013; Cardaropoli et al., 2014). According to the World Health Organization, maternal anemia is determined when the expectant woman’s Hb concentration goes below 11 g/dl (World Health Organization [WHO], 1994). Research has shown an association between moderate to severe anemia in pregnancy and a higher likelihood of adverse gestational outcomes, which includes underweight babies and premature deliveries (Cardaropoli et al., 2014; Azami et al., 2017). Pregnant women with *H. pylori* infection were found to have low Hb levels from the start of pregnancy (Weyermann et al., 2005). Muhsen (2013) advised testing for *H*. *pylori*, suggesting that it may be a factor causing anemia in pregnant women. This recommendation stems from several studies indicating a connection between *H. pylori* and anemia. In their meta-review of epidemiologic studies, interventional trials, case reports, and series, Muhsen and Cohen (2008) found iron deficiency anemia to occur more frequently in women with *H. pylori* than in uninfected women.

In the present study, Hb content was significantly decreased in cases with respect to controls. When related to *H. pylori*, Hb was significantly lower in infected pregnant women than in negative cases. Anemia was an observed complication of infection with *H. pylori*, and the positive correlation between anemia and *H. pylori* in expectant women has previously been documented. Weyermann et al. (2005) reported a reduction in Hb levels in early pregnancy in women with *H. pylori* compared to those without (-0.25 g/dl; 95% CI: -0.49 to -0.003). As the pregnancy progressed, this change in Hb level worsened (-0.14 g/dl; 95% CI: -0.38 to 0.10) (Weyermann et al., 2005). In their cross-sectional analysis of 117 expectant women, Mulayim et al. (2008) reported that 27 were anemic, and all of these women were infected with *H*. *pylori*, raising the risk of restricted fetal growth (Mulayim et al., 2008). A small-scale prospective study examining the HG–*H. pylori* relationship demonstrated that infected women suffering from HG have a higher risk of iron deficiency anemia than symptomatic uninfected women (Bezircioglu et al., 2011). In another case–control study, the authors found that anemia had a much higher prevalence in women with HG and HpSA than in those without HpSA or those in the control group (Abd Alwahed et al., 2014). Several possible explanations have been suggested for the connection between *H. pylori* infection and anemia: the direct outcome of *H. pylori* on iron absorption, reduction of vitamin C concentrations in the stomach, bleeding of gastric ulcers, or *H. pylori*’s capture and usage of iron (Ciacci et al., 2004; Cardenas et al., 2006; Muhsen and Cohen, 2008; Mubarak et al., 2014).

The liver is one of the organs that, according to some evidence, may be affected by *H. pylori* infection; however, the exact effects of the bacterial infection on hepatic function and the underlying mechanisms are still unclear (Suzuki et al., 2011). Considering the elevated rate of *H. pylori* infection in the general population and some evidence indicating a correlation between *H. pylori* infection and some degree of liver damage, we evaluated whether *H. pylori* has any effects on the level of liver enzymes in the pregnant women studied. The results indicated a significantly elevated mean level of liver enzymes in women in the case group compared to the controls. The activity of AST was significantly elevated in cases testing positive compared to that in negative cases, findings which are in line with reports from previous research (Graham et al., 1998). Considering no association of *H. pylori* with ALT level, which is more specific than AST, these authors introduced two hypotheses: that there is an extrahepatic source for increased AST level and/or that there is a host genetic predisposition to both *H. pylori* infection and increased levels of liver enzymes (Graham et al., 1998).

The majority of the research exploring a connection between *H. pylori* and HG was carried out using serologic tests (Frigo et al., 1998; Kocak et al., 1999; Salimi-Khayati et al., 2003; Mansour and Nashaat, 2011). Because seroconversion remains as long as months or even years after recovery, these tests cannot distinguish acute from chronic *H. pylori* infections (Gisbert et al., 2006). In the current study, we used the stool antigen test to detect the presence of the bacterium. A reliable non-invasive marker, this test picks up bacterial antigens of any current infection in the stool (Gisbert et al., 2006).

The primary strength of this study involves the use of the stool antigen test and the sample size. Due to the small sample size, the study was carefully done, and the results may be more accurate than those from larger studies. Of course, this small sample size is also the main limitation of this study. It means that although we found a significant correlation between HG and *H. pylori*, the power of this finding is low, so there may be actual sociodemographic differences between women with HG and those without that did not reach the level of significance. In addition, other potential confounders such as socioeconomic level and gestational or preexisting complications were not accounted for.

## Conclusion

Although a link between *H. pylori* and HG has previously been established, this paper makes an important contribution to the literature by confirming this relationship among Saudi women. In our study of pregnant women, we found a clear connection between *H. pylori* infection and HG, pointing to *H. pylori* as one contributing factor of this complication of pregnancy. *H. pylori* testing should be included in investigations of HG, especially when the condition does not respond to treatment and in cases continuing past the first trimester. Additional research on *H. pylori* infection in the population of pregnant women who are experiencing severe vomiting and nausea is warranted.

## Data Availability Statement

The raw data supporting the conclusions of this article will be made available by the authors, without undue reservation, to any qualified researcher.

## Ethics Statement

The studies involving human participants were reviewed and approved by Biomedical Ethics Research Committee at the Faculty of Medicine, King Abdulaziz University. The patients/participants provided their written informed consent to participate in this study.

## Author Contributions

The author contributed in project development, data collection and analysis, and manuscript writing and editing.

## Conflict of Interest

The author declares that the research was conducted in the absence of any commercial or financial relationships that could be construed as a potential conflict of interest.
